# A critical role of endothelial cell protein C receptor in the intestinal homeostasis in experimental colitis

**DOI:** 10.1038/s41598-020-77502-3

**Published:** 2020-11-25

**Authors:** Vijay Kondreddy, Shiva Keshava, Charles T. Esmon, Usha R. Pendurthi, L. Vijaya Mohan Rao

**Affiliations:** 1 Department of Cellular and Molecular Biology, The University of Texas Health Science Center At Tyler, 11937 US Highway 271, Tyler, TX 75708-3154 USA; 2 Coagulation Biology Laboratory, Oklahoma Medical Research Foundation, Oklahoma City, OK USA

**Keywords:** Gastroenterology, Pathogenesis

## Abstract

Crohn’s disease and ulcerative colitis are the two forms of disorders of the human inflammatory bowel disease with unknown etiologies. Endothelial cell protein C receptor (EPCR) is a multifunctional and multiligand receptor, which is expressed on the endothelium and other cell types, including epithelial cells. Here, we report that EPCR is expressed in the colon epithelial cells, CD11c^+^, and CD21^+^/CD35^+^ myeloid cells surrounding the crypts in the colon mucosa. EPCR expression was markedly decreased in the colon mucosa during colitis. The loss of EPCR appeared to associate with increased disease index of the experimental colitis in mice. EPCR^−/−^ mice were more susceptible to dextran sulfate sodium (DSS)-induced colitis, manifested by increased weight loss, macrophage infiltration, and inflammatory cytokines in the colon tissue. DSS treatment of EPCR^−/−^ mice resulted in increased bleeding, bodyweight loss, anemia, fibrin deposition, and loss of colon epithelial and goblet cells. Administration of coagulant factor VIIa significantly attenuated the DSS-induced colon length shortening, rectal bleeding, bodyweight loss, and disease activity index in the wild-type mice but not EPCR^−/−^ mice. In summary, our data provide direct evidence that EPCR plays a crucial role in regulating the inflammation in the colon during colitis.

## Introduction

Inflammatory bowel disease is a chronic, relapsing, and remitting disease with an unknown etiology^1^. The factors that trigger the inflammation in the immunocompromised colon are not fully identified, but it is believed that the loss of mucosal barrier function together with the activation of underlying immune cells initiates the disease^2,3^. Increasing evidence suggests that the nonimmune cells, including the colon epithelial cells, vascular cells, and platelets, significantly contribute to the mucosal homeostasis in the colon^4–6^. It is well-established that the dysfunction of microvasculature is critically involved in the colon inflammation^7,8^.

Endothelial cells selectively maintain the immune homeostasis by regulating the barrier permeability, leukocyte traffic, and the coagulation system that keeps the epithelial cells and immune cells in an unresponsive state in the colon^9^. During colon mucosal inflammation, endothelial cells regulate neutrophil, monocyte, and lymphocyte efflux into the intestinal space by upregulating cell-adhesion molecules and chemokine secretion^10^. During human IBD, the endothelium is activated, as evident with the aberrant expression of cell-adhesion molecules, infiltration of leukocytes, and upregulation of cytokines^11,12^.

EPCR is expressed highly in the endothelium lining blood vessels^13^. EPCR plays a critical role in the protein C anticoagulant pathway by binding to protein C and promoting the activation of protein C by thrombin-thrombomodulin (TM) complex^14^. Activated protein C (APC) inactivates two critical coagulation cofactors, FVIIIa and FVa, by proteolytic cleavage, and thus downregulates thrombin generation^15^. EPCR is also shown to play a key role in supporting APC-mediated cytoprotective effects through activation of protease-activated receptor (PAR)-mediated cell signaling^16–18^. Recent studies demonstrated that EPCR also binds other ligands, including coagulation protein factor VIIa (FVIIa), neutrophil proteinase-3, *Plasmodium falciparum* erythrocyte membrane protein 1 (pfEMP1), and T cell receptor (TCR) present on a subset of Vδ2^−^ γδ T cells^19^. These observations suggest that EPCR may influence various cellular functions in pathophysiology by coupling with different ligands^19^.

Recent studies demonstrate that the protein C-EPCR pathway governs the intestinal inflammation during IBD^20–22^. EPCR and TM were expressed on the mucosal endothelium, but their expression was decreased in IBD, which in turn caused impairment of protein C activation^20,23^. The downregulation of the protein C pathway was shown to correlate to the active disease during colitis clinically^20^. The APC treatment was shown to suppress mucosal barrier permeability and inflammation during experimental colitis^20,21^. Consistent with the above data, PC^−/−^/PC (low-PC) mice, expressing only 3% of protein C, were prone to severe experimental colitis^21^. Although the above studies demonstrate the importance of the EPCR-protein C pathway in the pathogenesis of IBD, owing to the presence of diverse ligands to EPCR, the use of protein C-deficient mice alone does not provide a full picture of the pathological significance of EPCR during colitis. In this context studies employing EPCR^−/−^ mice may provide more relevant and direct information on the consequences of the loss of EPCR in IBD in the progression of the disease.

In the present study, we confirm that EPCR is expressed in epithelial and leukocytes surrounding the crypts in the colon mucosa, and the loss of EPCR expression in experimental colitis is associated with an increased disease index. EPCR-deficient mice are highly susceptible to DSS-induced colitis. FVIIa treatment reduced the severity of the DSS-induced experimental colitis in wild-type mice but not ECR^−/−^ mice.

## Materials and methods

### Materials

Dextran sulfate sodium was purchased from Affymetrix (ThermoFisher Scientific, Waltham, MA, USA). ELISA kits for IL-1β, MCP-1, and IL-6, and CD11c (N418)-PE conjugated antibodies were obtained from eBioscience (ThermoFisher Scientific). Rat CD21/CD35 AF-594 conjugated antibody was purchased from BioLegend, CA. The antibodies against mouse EPCR were described elsewhere^24^. The antibodies against fibrin and F4/80 were from EMD Millipore (Burlington, MA, USA). Hemoccult Sensa fecal occult blood test kit was from Modomed (Grand Rapids, MI, USA). rhFVIIa was a gift from the late Dr. Walter Kisiel, School of Medicine, The University of New Mexico, Albuquerque, NM, USA.

### Mice

The generation of EPCR^−/−^ mice (Procr^−/−^) was described previously^25^. Wild-type C57BL/6 mice were generated from the in-house breeding program or obtained from Jackson Laboratory (Bar Harbor, ME).

### DSS induced colitis in the mice

All animal studies reported herein were approved by the Institutional Animal Care and Use Committee at the University of Texas Health Science Center at Tyler, TX, USA. Animal husbandry and experiments were conducted according to the animal welfare guidelines outlined in the Guide for the Care and Use of Laboratory Animals. Colitis was induced in mice by giving 2.5% DSS (w/v) dissolved in the sterile drinking water ad libitum for 10 days. Control mice received sterile drinking water with no DSS. The food and water intake of the mice were monitored daily throughout the experimental period (10 days). The severity of the disease was assessed based on the clinical symptoms of colitis, such as body weight loss, stool consistency, and blood in the stools. A fecal occult blood test kit was used to measure fecal blood according to the manufacturer’s instructions. All three parameters were weighted equally. The scoring as followed, bodyweight loss, 0–4 (0 = < 1%, 1 = 1–5%, 2 = 5–10%, 3 = 10–15%, and 4 = > 15%), stool consistency, 0–4 (0, normal; 2, soft; 4, diarrhea), and stool blood, 0–4 (0, no blood; 2, low to moderate levels; 4, high levels). Disease activity index (DAI) was calculated using the sum of the values of body weight loss, diarrhea, and stool blood, with a maximum score of 12 for severe colitis^26,27^. After 10 days of colitis, an aliquot of blood was collected for the measurement of hemoglobin in peripheral blood. rhFVIIa was administered to the mice intravenously via the tail vein at a dose of 250 µg/kg b.w on alternative days, initiated at day 1 of feeding with DSS water. The animals were killed by exsanguination, the colon tissue was excised, and the colon length was measured. The distal portion of the colon was either fixed in the EXCELL fixative (Stat Lab, McKinney, TX, USA) or frozen in Optimal Cutting Temperature (OCT) compound (Sakura Finetek USA Inc., Torrance, CA) for histological and immunohistochemical analysis. Some of the samples were also stored at − 80 °C for the measurement of cytokines later.

### Colon myeloperoxidase (MPO) assay

MPO activity was determined by a dianisidine-H_2_O_2_ method, as described earlier^27^. Colon tissue samples were homogenized in potassium phosphate buffer (0.05 M, pH 6.0) containing 0.5% hexadecyltrimethylammonium bromide. The homogenates were centrifuged at 10,000 × *g* for 20 min at 4 °C. An aliquot of the supernatant was added to the reaction mixture containing 2910 μl of 50 mM phosphate buffer (pH 6.0), 30 μl of *O*-dianisidine dihydrochloride (20 mg/ml), and 30 μl of 20 mM H_2_O_2_. The reaction was terminated after 10 min by adding 30 μl of 2% sodium azide. The change in the absorbance was measured at 460 nm using a spectrophotometer. 1 U of the enzyme was arbitrarily defined as the amount of enzyme that produces a change in absorbance of 1.0/min.

### Analysis of blood hemoglobin

The blood was collected via submandibular vein puncture into a citrate anticoagulant. Hemoglobin (Hb) was extracted by adding 10 μl of blood to 1 ml of ABX Lysebio solution (Horiba Medical, Irvine, CA, USA) for at least 2 h and the absorbance was read in the spectrophotometer at 550 nm. Hemoglobin was also extracted from the known volumes of freshly collected mouse blood to generate a standard curve. The concentration of hemoglobin in healthy mouse blood was taken as 14 g/dl. The Hb levels from experimental mice were determined from extrapolation of the absorbance values from the standard curve^28^.

### Colon cytokine analysis

The colon tissues from the mice were snap-frozen in liquid nitrogen, and the frozen tissue was pulverized into powder. The powder was suspended in RIPA buffer (Pathscan, CST, USA) containing protease inhibitors. The lysate was briefly sonicated and centrifuged at 10,000 × *g* for 20 min at 4 °C. IL-6, IL-1β, and MCP1 levels in tissue extracts were estimated using ELISA kits (eBioscience).

### Histopathology

The colon tissues were fixed in EXCELL fixative for 24 h or more and processed for embedding in paraffin. Thin tissue sections (5 μm) were cut and stained with hematoxylin and eosin, and the stained tissue sections were observed under a light microscope (Olympus BX41, PA) to assess microscopic damage and extravasation of leukocytes. Images were captured with an Olympus DP25 camera. The epithelial damage, infiltration, and histology were evaluated and scored as described previously^29,30^. Briefly, epithelial damage was scored 0 to 4 (0, normal colon architecture; 1, loss of a few goblet cells; 2, loss of goblet cells in large areas; 3, loss of a few crypts; 4, loss of crypts in large areas). Leukocyte infiltration was also scored 0–4 (0, no infiltration; 1, infiltration around crypts; 2, infiltration reaching to muscularis mucosae; 3, extensive infiltration and thickening of the mucosa with edema; 4, infiltration into the submucosa). The total histological score represents the sum of the epithelial damage and the infiltration score.

### Immunohistochemistry and immunofluorescence microscopy

For immunohistochemistry, the antigen retrieval was done by boiling tissue sections for 15 min in sodium citrate buffer (10 mM, pH 6.0). Endogenous peroxide activity was quenched by incubating tissue sections with 3% hydrogen peroxide. After blocking the tissue sections with an antibody diluent containing background reducing components (Agilent, Santa Clara, CA), they were incubated with control IgG, primary antibodies against EPCR, or fibrin (5 µg/ml) for overnight at 4 °C. The sections were then incubated with biotin-labeled goat anti-rat IgG (1:500) and ultrasensitive streptavidin-HRP (1:500) (Sigma, St. Louis, MO, USA) and developed using AEC-hydrogen peroxide substrate solution. The sections were counterstained with hematoxylin, mounted, and visualized with an Olympus BX41 microscope.

For immunofluorescence microscopy, the colon tissues were cut open longitudinally and frozen in Optimal Cutting Temperature compound for sectioning. Thin (5 µm) sections were cut, and the sections were rehydrated in PBS and blocked in 10% FBS. The tissue sections were incubated with rat anti-murine F4/80, goat anti-murine EPCR, hamster anti-murine CD11c-PE, rat anti-mouse CD21/CD35-AF594 antibodies, or control isotype IgG (10 µg/ml) overnight at 4 °C. After washing the sections thrice with PBS, they were incubated with AF488-conjugated donkey anti-rat IgG or AF488-conjugated donkey anti-goat IgG for 90 min. The sections were stained with DAPI to visualize the nucleus. The sections were mounted using Fluro-Gel (EMS, Hatfield, PA, USA) and analyzed by confocal microscopy (LSM 510, Zeiss).

### Data analysis

The data were shown as mean ± SEM. Normality tests, Shapiro–Wilk was used to determine if the data were normally distributed. Since most of the data were passed the normality test, parametric tests were used to calculate statistical significance. Student’s t-test was used to calculate statistical significance between the two groups. *p* < 0.05 was considered as statistically significant. Graphpad Prism version 8 was used for statistical analysis and preparation of figures.

## Results

### DSS injury downregulates mucosal EPCR expression in the colon

**Figure 1:**
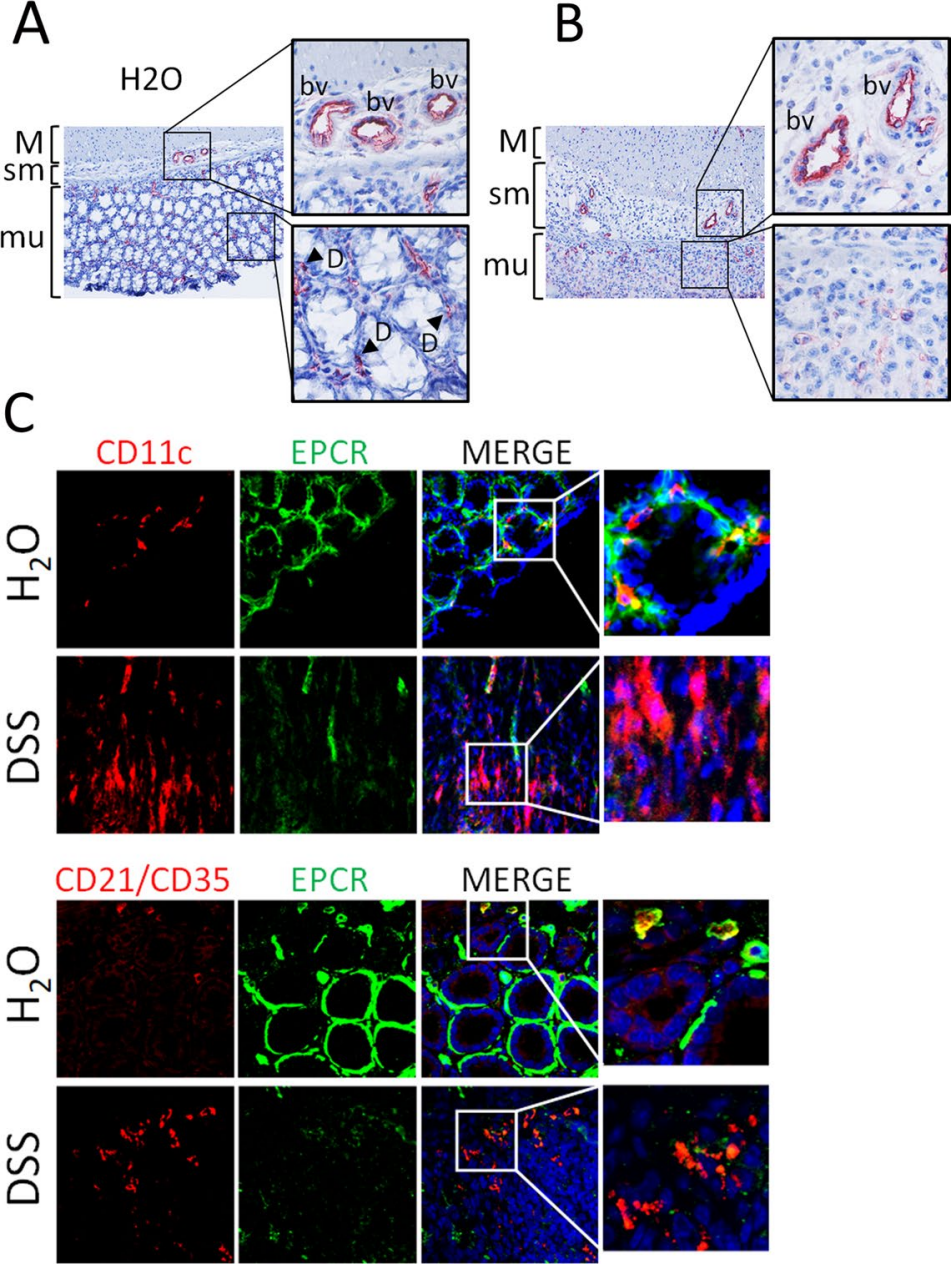
DSS-induced colitis results in the loss of EPCR in the colon mucosa and not in endothelial cells lining blood vessels. Wild-type (C57BL/6J) mice were fed with control drinking water (H_2_O) or drinking water containing DSS (2.5% w/v) for 10 days. At the end of 10 days, the colon tissues were collected from the mice and processed for immunohistochemistry using goat anti-mouse EPCR antibodies. Tissue sections were imaged at 20X magnification, and boxed areas were digitally magnified (**A**) colon tissue section from control mice; (**B**) colon tissue section from DSS-treated mice. Red staining indicates EPCR positivity. M, muscularis externa; sm, submucosa; mu, mucosa; bv, blood vessels, L, Leukocytes. Arrows in panel A point out leukocytes. (**C**) The colon tissue sections were immunostained for EPCR (green), CD11c (red), or CD21/35 (red). Nuclei were stained with DAPI (blue). Boxed areas were digitally enlarged. Yellow color in merged images indicates the colocalization of EPCR with CD11c or CD21/35.

Analysis of EPCR expression in the colon of wild-type (C57BL/6J) mice by immunohistochemistry revealed, as expected, EPCR expression on the endothelium lining blood vessels of the mucosa and sub-mucosa of the colon. In addition to the endothelium, we also found EPCR expression in cells surrounding the colon crypts in the lamina propria of the mucosa (Fig. 1A). Earlier studies suggested that these cells were colon epithelial cells and dendritic-like cells^21,23^. EPCR expression in epithelial cells was weak compared to EPCR expression in endothelial cells and thus not fully evident in immunohistochemistry images. However, immunofluorescence confocal microscopy confirmed that colon epithelial cells express EPCR as a characteristic pattern surrounding crypts (Fig. 1C). To identify the leukocytes that express EPCR in the colon tissue, we have performed dual immunostaining with antibodies against EPCR and CD11c or CD21/CD35. Earlier studies indicated that CD11c^+^ and CD21/CD35^+^ cells were dendritic-like cells^23,31–33^. We found expression of EPCR in CD11c^+^ and CD21/CD35^+^ cells in the colon of uninjured control mice (Fig. 1C). The colon histology showed that DSS treatment resulted in the substantial loss of the crypt architecture and goblet cells in the severely inflamed regions of the colon. The colon mucosa was infiltrated by cells, probably leukocytes in DSS-treated animals (Fig. 1B). The number of CD11c^+^ and CD21/CD35^+^ cells increased in the colon following DSS treatment, indicating infiltration of CD11c^+^ and CD21/CD35^+^ cells into the colon in colitis. DSS treatment had no significant effect on the EPCR expression in the endothelium (Fig. 1B). However, DSS-treatment significantly downregulated the EPCR expression in colon epithelial cells, CD11c^+^, and CD21/CD35^+^ cells (Fig. 1C). Since no EPCR expression was detected almost in all CD11c^+^ and CD21/CD35^+^ cells in the colon of DSS-treated mice, it indicates the loss of EPCR expression in resident CD11c^+^ and CD21/CD35^+^ cells. It is unknown at present whether infiltered CD11c^+^ and CD21/CD35^+^ cells lack EPCR to start with or lose their expression following the infiltration into the colon in DSS-treated mice.

### Increased susceptibility of EPCR^−/−^ mice to DSS-induced colitis

**Figure 2:**
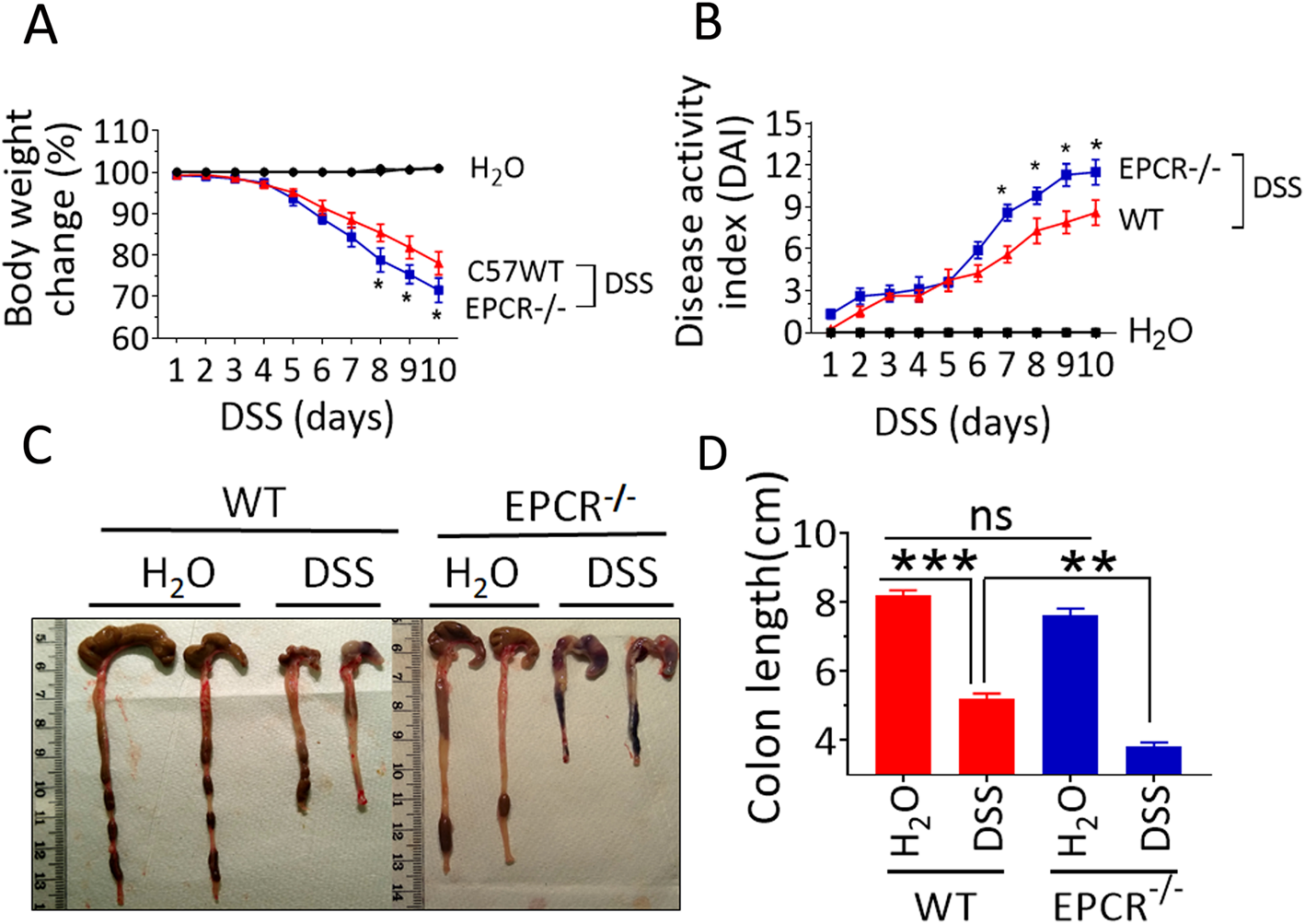
EPCR deficiency exacerbates the DSS-induced colitis in mice: Wild-type and EPCR^−/−^ mice were treated with DSS (2.5% w/v) in the drinking water for 10 days. The disease activity index (DAI) was monitored during the course of colitis based on body weight loss, diarrhea, and fecal blood. The body weight loss (**A**) and DAI (**B**) were calculated as described in Methods. (**C**) Representative pictures of the colon from the control (H_2_O) and DSS-treated mice. (**D**) The colon length of control and DSS-treated mice. Data are the mean ± SEM of 2 independent experiments of 8 mice/group. *P < 0.05; **P < 0.01; ***P < 0.001.

Both wild-type and EPCR^−/−^ mice were treated with DSS (2.5% in the drinking water) for 10 days to induce colitis. The DSS-induced colitis was evaluated by body weight loss, diarrhea, and rectal bleeding. Both the wild-type and EPCR^−/−^ mice receiving DSS exhibited severe symptoms of colitis, including the loose stools starting between day 3 and day 5, and bloody diarrhea after day 5. Both groups of mice lost bodyweight, starting from day 3 of DSS treatment (Fig. 2A). However, the colitis in the EPCR^−/−^ mice was more severe compared to wild-type mice, as evidenced by more weight loss, diarrhea, and increased anal bleeding in EPCR^−/−^ mice compared to the wild-type mice. It may be pertinent to point out here that there were no significant differences in the body weight between wild-type and EPCR^−/−^ mice at the time of initiation of DSS treatment. Evaluation of the severity of colitis by monitoring the disease activity index (DAI) showed that there were no significant differences between wild-type and EPCR^−/−^ mice until day 6. After day 6, the colitis was significantly (*p* < 0.05) severe in EPCR^−/−^ mice compared to wild-type mice (Fig. 2B). It is known that DSS-treatment in mice reduces the colon length, and the colon length shortening is often considered as a good marker of the disease^34^. As shown in Fig. 2C, DSS treatment reduced the length of the colon in both wild-type and EPCR^−/−^ mice, compared to their controls not fed with DSS. The colon length shortening was significantly higher (*p* < 0.01) in the DSS-treated EPCR^−/−^ mice compared to the DSS-treated wild-type mice. Furthermore, blood was seen visually in the colon tissues of EPCR^−/−^ mice fed with DSS, which was not present in DSS-treated wild-type mice. There were no significant differences in the colon length between wild-type and EPCR^−/−^ mice that were fed with control drinking water (Fig. 2D).

### Increased mucosal damage and macrophage infiltration in EPCR^−/−^ mice

**Figure 3:**
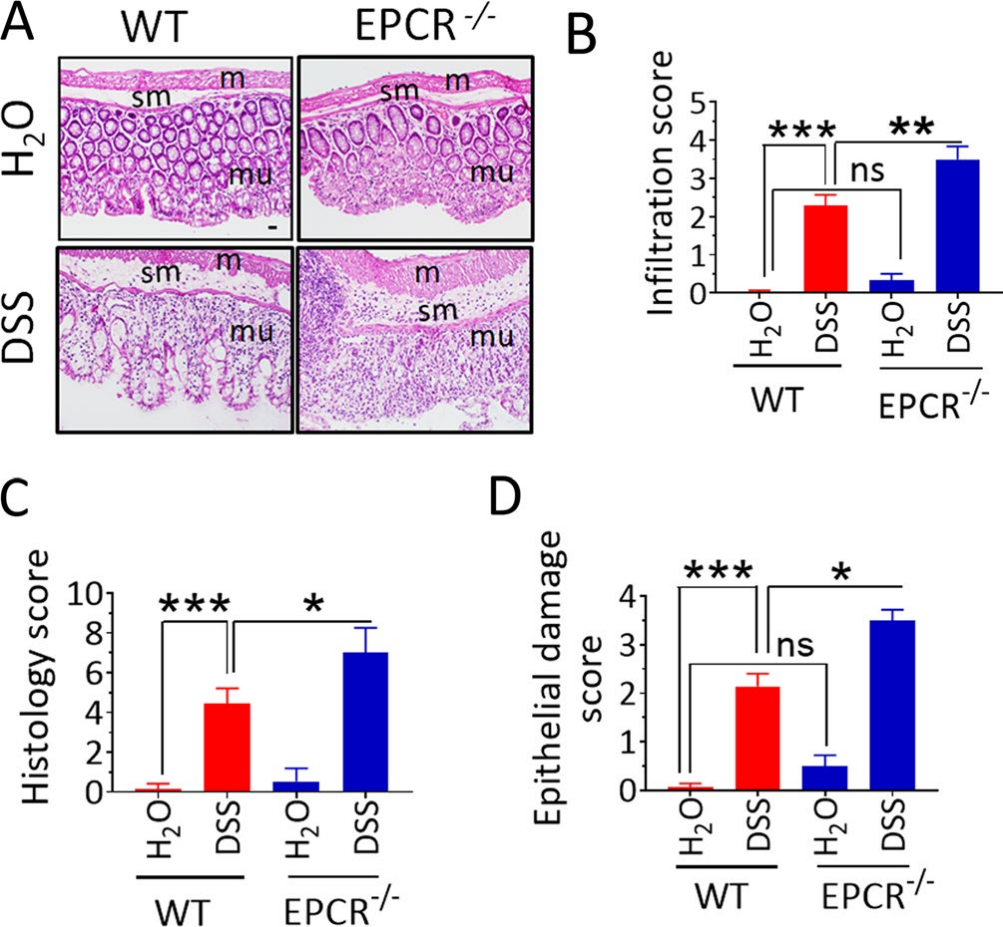
Histopathology of DSS-induced colitis in wild-type and EPCR^−/−^ mice. The colon tissues collected from the control and DSS-treated (2.5% w/v in the drinking water for 10 days) mice were processed for tissue sectioning and staining with H&E stain. Epithelial damage, infiltration, and histology were scored as described in Methods. (**A**) Representative images of H&E stained colon tissue sections. Sections were imaged at 10X magnification. The scale bar, 80 µm. m, muscularis externa; sm, submucosa; mu, mucosa. (**B**) Infiltration score; (**C**) Histology score; and (**D**) Epithelial damage score. Data are the mean ± SEM of 2 independent experiments of 8 mice/group. *P < 0.05; **P < 0 .01; ***P < 0.001; ns, no statistically significant difference.

DSS treatment resulted in the loss of goblet cells, distortion of crypts, mucosal ulceration, and increased leukocyte infiltration in the colon tissues of both wild-type and EPCR^−/−^ mice (Fig. 3A). However, notable differences were found in the histopathology of the colon tissues harvested from DSS-fed wild-type and EPCR^−/−^ mice. The ulceration and crypt distortion was much severe in the DSS-treated EPCR^−/−^ mice. Furthermore, the crypt denuded mucosal surface was heavily infiltrated by leukocytes in EPCR^−/−^ mice (Fig. 3A). We also observed an increased infiltration of leukocytes in the sub-mucosal area of the DSS-treated EPCR^−/−^ mice compared to the DSS-treated wild-type mice (Fig. 3A). There was a significant enlargement or thickening of the muscularis layer in the DSS-treated EPCR^−/−^ mice. Overall, the histology score, which was the summation of epithelial damage and leukocyte infiltration, was significantly (*p* < 0.05) higher in the EPCR^−/−^ mice (Fig. 3B–D). In control mice not fed with DSS, we found some minor differences in the colon architecture between wild-type and EPCR^−/−^ mice. The number of mucous producing goblet cells appears to be fewer in the crypts of EPCR^−/−^ mice. There is a slight distortion in the crypt architecture. However, we found no evidence of subclinical gastrointestinal disease in control EPCR^−/−^ mice.

**Figure 4:**
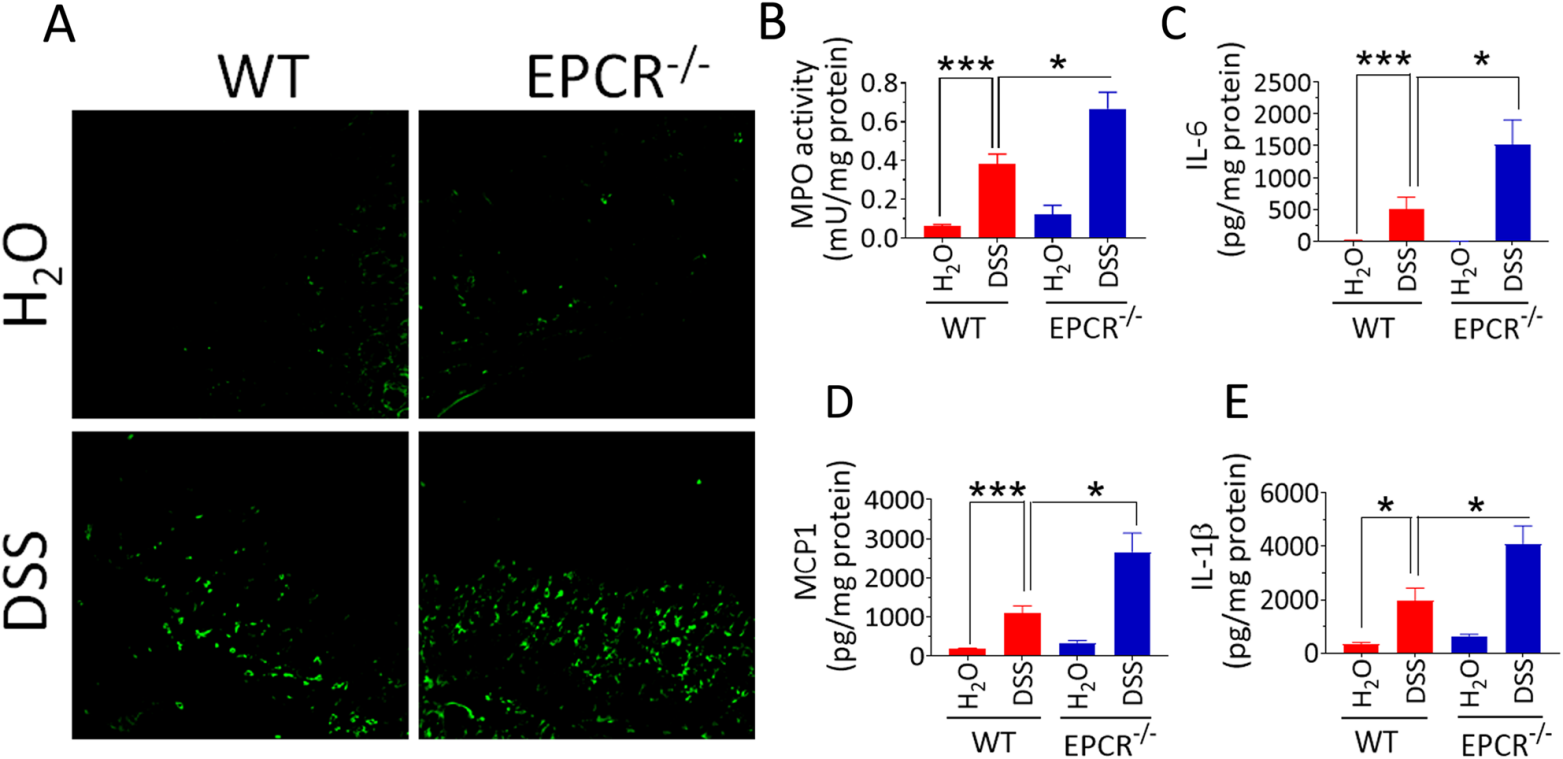
EPCR deficiency aggravates the DSS-induced macrophage infiltration and cytokine secretion in the colon. The colon tissues were collected from the control and DSS-treated mice and processed for tissue sectioning and preparing tissue extracts. (**A**) The colon tissue sections were stained with F4/80 antibody to identify macrophages by immunofluorescence microscopy. Green fluorescence spots indicate macrophages. Colon tissue extracts were used to measure myeloperoxidase activity (**B**), IL-6 (**C**), MCP1 (**D**), or IL-1ß (**E**). Data are the mean ± SEM of 2 independent experiments of 8 mice/group. *P < 0.05; ***P < 0.001.

Next, to assess the leukocyte infiltration in the colon, we stained the colon tissue sections for macrophages using F4/80 antibody, and the staining was evaluated by immunofluorescence microscopy. In agreement with the histopathological findings, the macrophage infiltration was elevated after DSS treatment in both the wild-type and EPCR^−/−^ mice. However, macrophage recruitment appears to be more pronounced in the EPCR^−/−^ mice compared to the wild-type mice (Fig. 4A).

### Increased myeloperoxidase activity and cytokine levels in EPCR-deficient mice

Myeloperoxidase (MPO) is produced by the infiltrated neutrophils and macrophages and is a marker of leukocyte infiltration during inflammation^35^. Analysis of MPO activity in the colon tissue showed a four-fold increase in the activity in the DSS-treated wild-type mice compared to the control mice. MPO activity levels were significantly (*p* < 0.05) elevated in the DSS-treated EPCR^−/−^ mice compared to its counterpart wild-type mice (Fig. 4B). Analysis of inflammatory cytokines IL-6, IL-1ß, and MCP-1 showed a marked increase in their levels in DSS-treated mice. The levels of inflammatory cytokines were significantly (*p* < 0.05) higher in DSS-treated EPCR^−/−^ mice compared to DSS treated wild-type mice (Fig. 4C–E).

### Increased blood loss, anemia, and fibrin/fibrinogen deposition in EPCR-deficient mice

**Figure 5:**
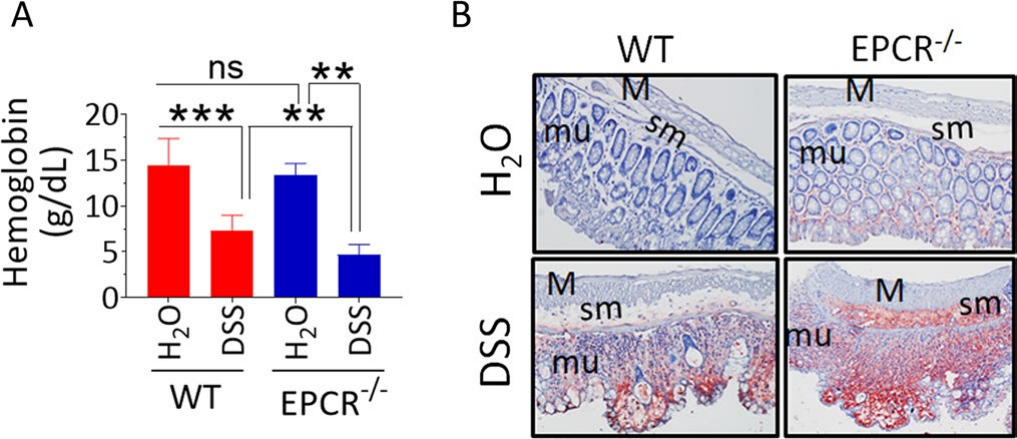
EPCR-deficient mice exhibit increased DSS-induced bleeding and fibrin/fibrinogen deposition in the colon. Blood and colon tissue samples were collected from the control and DSS-treated mice. (**A**) Blood samples were used to measure hemoglobin content. (**B**) The colon tissue sections were stained with fibrin/fibrinogen antibody overnight, followed by biotinylated secondary antibody and streptavidin-HRP. The sections were developed using the AEC substrate. The sections were counterstained with hematoxylin and imaged at 10X magnification. Red staining indicates positivity for fibrin/fibrinogen. m, muscularis externa; sm, submucosa; mu, mucosa. Data are the mean ± SEM of 2 independent experiments of 8 mice/group. *P < 0 .05; **P < 0 .01; ***P < 0.001.

As noted in the earlier section, the perianal bleeding due to DSS-induced colitis appeared to be more severe in EPCR^−/−^ mice compared to wild-type mice. To confirm and strengthen these findings, we analyzed hemoglobin levels in the peripheral blood as a marker of bleeding and anemia. DSS treatment induced severe anemia in wild-type mice, which was evident as an approximately 40% decrease in hemoglobin levels in the blood. The reduction in blood hemoglobin was much more severe in the DSS-treated EPCR^−/−^ mice (Fig. 5A). We also measured thrombin:antithrombin (TAT) complex levels in the plasma as a marker of ongoing coagulation. No significant differences were found in TAT levels between wild-type and EPCR^−/−^ mice and between healthy control and DSS-treated mice (wild-type control, 11.8 ± 1.2; DSS treated wild-type, 9.7 ± 1.7; EPCR^−/−^, 12.5 ± 2.1; and DSS treated EPCR^−/−^, 13.2 ± 1.1 ng/ml).

Next, we evaluated coagulation status at the tissue level by monitoring fibrin deposition in the colon tissues by immunohistochemistry. We observed a slight increase in the fibrin staining in control EPCR^−/−^ mice compared to control wild-type mice. DSS treatment markedly increased the fibrin deposition in the colon mucosal surface in wild-type mice (Fig. 5B). We also observed a slight increase in fibrin deposition in the sub-mucosal area in the DSS-treated wild-type mice. No positive fibrin staining was detected in the colon muscularis layer. In DSS treated EPCR^−/−^ mice, the fibrin staining was more intense in the denuded mucosal surface. We found a marked increase in fibrin deposition in the sub-mucosal area in the DSS-treated EPCR^−/−^ mice compared to DSS-treated wild-type mice (Fig. 5B).

### FVIIa treatment reduces the severity of DSS-induced colitis in an EPCR-dependent fashion

**Figure 6:**
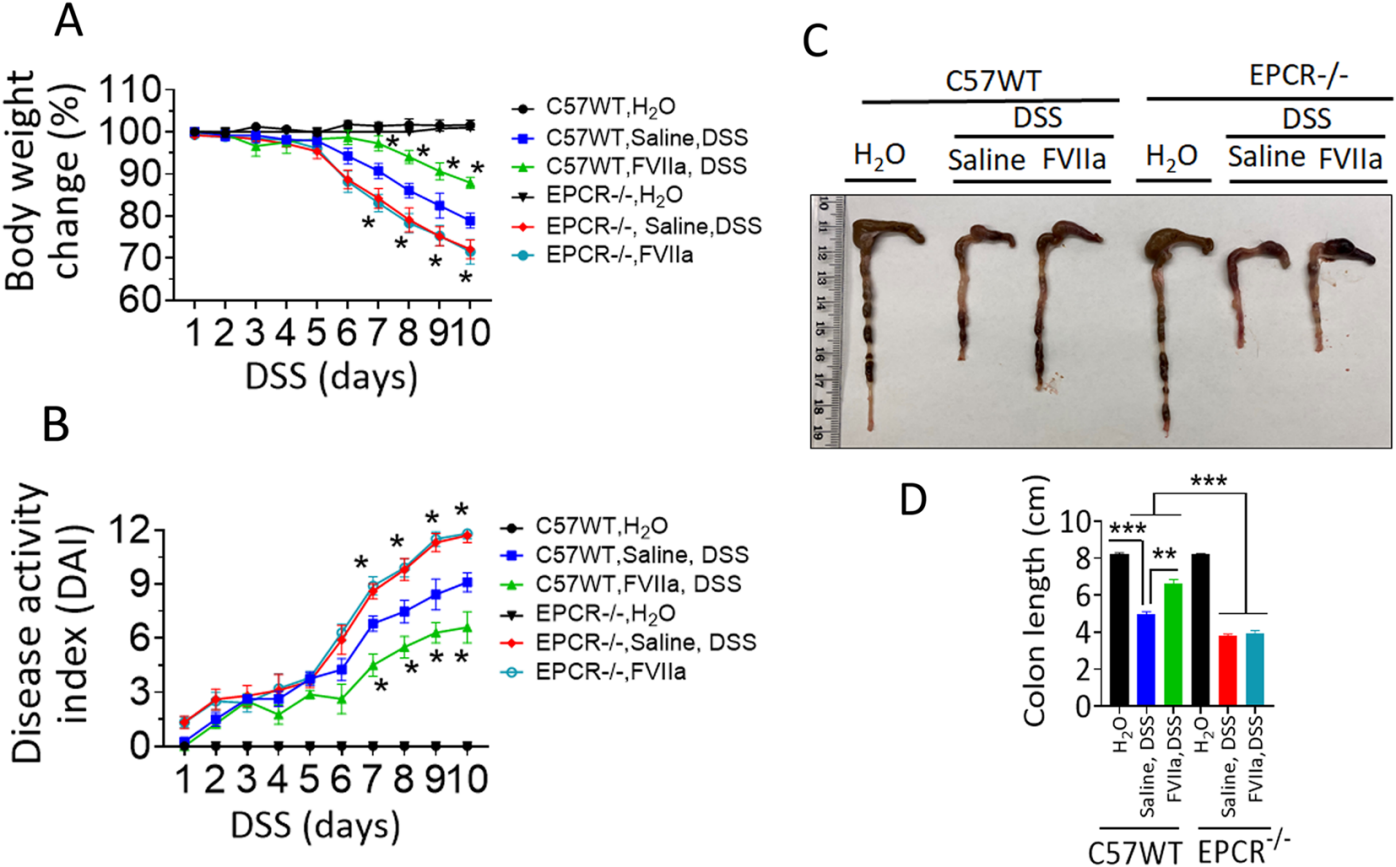
Pharmacological doses of FVIIa protects against intestinal bleeding, bodyweight loss, and disease activity in DSS-induced colitis in wild-type but not EPCR^−/−^ mice. Wild-type or EPCR^−/−^ mice were administered with a control vehicle (saline) or recombinant hFVIIa (250 µg/kg) every alternate day during the 10-day course of colitis, starting at day 1 of DSS-treatment. The body weight loss (**A**) and disease activity index (**B**) were measured. (**C**) Representative images of the colon collected from control and FVIIa treated mice. (**D**) The colon length measurements. Data are the mean ± SEM of 2 independent experiments of 8 mice/group. In panels A and B, data of DSS-fed wild-type mice were compared with FVIIa-treated DSS-fed wild-type mice, DSS-fed EPCR^−/−^ mice, or FVIIa-treated DSS-fed EPCR^−/−^ mice to determine statistical significance. *P < 0.05; **P < 0.01; ***P < 0.001.

FVIIa is a hemostatic agent, which is successfully being used to treat hemophilia patients with inhibitors^36^. Our recent studies demonstrated that FVIIa binding to EPCR protects against LPS-induced inflammation and endothelial barrier disruption^37,38^. Therefore, we investigated here whether FVIIa treatment protects against inflammation-induced colitis. As shown in Fig. 6A, FVIIa treatment significantly (*p* < 0.05) reduced body weight loss associated with DSS-induced colitis in the wild-type mice. We also observed a significant decrease in stool blood in FVIIa-treated wild-type mice. The overall disease activity index was significantly (*p* < 0.05) lower in wild-type mice treated with FVIIa (Fig. 6B). Analysis of colon length showed that DSS-induced colon length shortening was significantly (*p* < 0.01) lower in the FVIIa-administered wild-type mice (Fig. 6C, D). In contrast, FVIIa treatment failed to protect the DSS-induced bleeding, bodyweight loss, and colon macroscopic changes in the EPCR^−/−^ mice (Fig. 6A–D). As noted in the earlier sections, DSS-induced colitis significantly severe in EPCR^−/−^ compared to wild-type mice. These data clearly suggest that FVIIa induced anti-inflammatory benefits in colitis require EPCR.

## Discussion

The results of the present study demonstrate that EPCR^−/−^ mice were more susceptible to chemically induced experimental colitis. This was evident as there was severe diarrhea, anemia, and loss of body weight in the EPCR^−/−^ mice compared to wild-type mice subjected to DSS treatment to induce colitis. Furthermore, the distortion of colon architecture, leukocyte infiltration, and fibrin deposition in EPCR^−/−^ mice were more pronounced in DSS-induced colitis. These data suggest that EPCR plays a role in regulating inflammation in the colon.

The gut homeostasis is maintained by the intestinal barrier, which precludes the direct contact and activation of underlying immune cells from the luminal microbiota^1^. Furthermore, the colon microvascular endothelial barrier selectively regulates the leukocyte traffic and prevents their movement out of the circulation^9^. The PC-EPCR pathway tightly regulates both the above processes^20,21^. Earlier studies demonstrated that progressive loss of PC-EPCR pathway in gastrointestinal disorders, including CD and UC patients, correlates with the inflammation and severity of the disease^21,23^. Our present data show that colon epithelial cells, CD11c^+^, and CD21/C35^+^ myeloid cells surrounding the colonic crypts in the mucosa express EPCR. DSS treatment resulted in the loss of EPCR in the colon epithelial cells and also CD11c^+^ and CD21/C35^+^ myeloid cells. These data were consistent with the earlier findings that showed loss of EPCR expressing CD21^+^ dendritic-like cells in the mucosa of the colon from IBD patients^23^. Earlier studies showed that CD11c was expressed by macrophages and dendritic cells in the colon^39,40^. CD21/CD35 was shown to express on immature B cells and follicular dendritic cells in the colon^33,41,42^. At present, it is unclear whether CD11c^+^ and CD21/C35^+^ myeloid cells in the colon observed in the present study are dendritic cells or other types of myeloid cells. Interestingly, we found an increased number of CD11c^+^ and CD21/35^+^ cells in the DSS-treated mice. It is likely that the increased number of CD11c^+^ and CD21/35^+^ cells in the colon of DSS-treated mice represent the infiltration of immune cells, including macrophages and neutrophils. Both monocytes/macrophages and neutrophils were shown to express EPCR^43–45^. Therefore, loss of EPCR expression in CD11c^+^ and CD21/C35^+^ cells in the colon of DSS-treated mice indicates the downregulation of EPCR expression in both resident and infiltered immune cells in colitis. In contrast to earlier data noted with colon tissues from IBD patients^20,23^, we found no decrease in endothelial EPCR in DSS-induced colitis. This could reflect potential differences between the pathogenesis of IBD in humans and the DSS-induced experimental colitis in mice. Further studies are needed to confirm the identity and significance of EPCR^+^ CD11c^+^ and CD21/C35^+^ cells in intestinal homoestasis.

EPCR is a multi-ligand and multifunctional receptor that is abundantly expressed in vascular endothelial cells. Upon engagement with its ligand protein C, EPCR promotes the activation of protein C-mediated anticoagulant pathway and elicits anti-inflammatory and barrier protective effects through EPCR-APC-PAR1-mediated signaling^16,17,19,46^. EPCR was shown to suppress inflammation by inhibiting the expression of inflammatory cytokines, chemokines, and cell-adhesion molecules during inflammation^19,37^. Our present data show that the loss of EPCR in the colon exacerbates the disease activity by upregulating inflammation during colitis. Since EPCR is the key player in the anticoagulant pathway, one would anticipate that the loss of EPCR or EPCR deficiency would lead to less intestinal bleeding upon DSS treatment. Contrary to this expectation, we observed increased perianal bleeding and severe anemia in the EPCR^−/−^ mice subjected to DSS treatment. It is possible that increased inflammation and vascular permeability in EPCR^−/−^ mice could be responsible for increased bleeding in these mice in DSS-induced colitis.

In earlier studies, Vetrano et al.^21^ used mice expressing very low levels of protein C (PC) to investigate the role of the PC-EPCR pathway in intestinal inflammation in DSS-induced colitis. Our present studies with EPCR^−/−^ mice support the conclusions reached in the earlier study that the loss of the PC-EPCR pathway exacerbates DSS-induced experimental colitis. However, in contrast to the earlier study with low-PC animals^21^, we found no evidence in the present study that EPCR deficiency leads to the development of spontaneous intestinal inflammation. Our present data were consistent with our earlier study, which showed no evidence for spontaneous inflammation in other organs, such as lung and kidney, in EPCR-deficient mice^37^. It was believed that spontaneous colitis observed in low-PC mice stems from spontaneous elevated levels of inflammatory cytokines and reduced or altered expression of tight junction proteins in the intestine in these mice^21^. We found no significant differences in cytokine levels between wild-type and EPCR^−/−^ mice in basal conditions (not subjected to DSS treatment). At present, it is unclear why the loss of the PC-EPCR pathway affects differently in developing spontaneous colitis in low-PC and EPCR^−/−^ mice. EPCR promotes the activation of PC to APC by thrombin/thrombomodulin^14^. In the absence of EPCR, low levels of APC could be generated in vivo^25,47^. Therefore, the levels of APC would be likely to be higher in EPCR^−/−^ mice compared to low-PC mice. The potential differences in basal levels of APC in low-PC and EPCR^−/−^ mice could explain differences between them in developing spontaneous colitis. Although we have not observed spontaneous inflammation or colitis in EPCR^−/−^ mice, we found a slightly disturbed crypt architecture in EPCR^−/−^ mice. These minor alterations in EPCR^−/−^ mice may not be sufficient to trigger spontaneous colitis, but could predispose them to readily develop colitis following minor perturbations in intestinal homeostasis.

The coagulation and inflammation are the two interconnected processes where the upregulation of one pathway influences the other^48^. The fibrin and thrombin are the key mediators that are generated during coagulation. They were shown to induce the expression of cell-adhesion molecules and cytokines, which play important roles in recruiting leukocyte in inflammation^49^. Because increased coagulation and thrombosis are well-documented events in IBD^49,50^, it would be reasonable to hypothesize that loss of EPCR would elevate fibrin deposition and increase TAT levels in colitis. Consistent with this hypothesis, EPCR deficiency resulted in increased fibrin deposition in the denuded surface of the colon mucosa and also the submucosal area in the DSS-treated mice. However, we found no significant increase in TAT levels in EPCR^−/−^ mice subjected to DSS treatment. These data indicate that DSS-induced inflammation is localized to the colon and not systemic. The increased fibrin(ogen) deposition in the colon in EPCR^−/−^ mice could have stemmed from multiple mechanisms. EPCR-deficiency could exacerbate endothelial dysfunction, barrier permeability, inflammation, and bleeding during colitis. As a result, the fibrinogen might get extravasated and deposited into the perivascular mucosal and sub-mucosal region. Alternatively, the attenuation of the anticoagulant pathway in EPCR^−/−^ mice could enhance the coagulation system, which ultimately leads to enhanced fibrin generation.

At present, it is unclear mechanisms by which EPCR deficiency exacerbates the severity of experimental colitis in mice. EPCR deficiency impairs the APC anticoagulant pathway and APC- and FVIIa-mediated cytoprotective signaling. EPCR deficiency could also alter other cellular processes as EPCR shown to interact with other ligands^19^. Impairment in both anticoagulant and cytoprotective activities could be responsible for the increased severity of the disease in EPCR^−/−^ mice. However, the anticoagulant activity of EPCR may play a predominant role in the pathogenesis of colitis as the enhanced activation of coagulation in EPCR^−/−^ mice could drive inflammation^48,51^. It is unlikely that impairment in endogenous APC- or FVIIa-mediated cytoprotective signaling plays a major role in exacerbating the colitis in EPCR^−/−^ mice because their levels may be too low to activate cytoprotective signaling pathways. Furthermore, murine FVIIa, unlike human FVIIa, does not bind EPCR^52^.

FVIIa is an approved hemostatic drug to treat hemophilia patients with inhibitors^53–55^. Several off-label therapies using FVIIa were proved to be beneficial in treating life-threatening disorders, including intracranial hemorrhage and trauma^56–59^. However, rFVIIa off-label treatment was shown to associate with a slightly enhanced risk for thrombotic complications, particularly in older patients and patients with underlying cardiovascular diseases^55,60^. Studies from our laboratory established that FVIIa binds EPCR with a similar affinity as of protein C and APC^61^. We also showed that FVIIa induces barrier protective and anti-inflammatory effects through the EPCR-PAR1-β-arrestin1 axis^37^. The present study shows that FVIIa treatment may reduce the severity of inflammation-induced colitis. The excessive bleeding that occurs during colitis induces an inflammatory response that appears to play a central role in colitis^62^. However, it is unlikely that the hemostatic effect of FVIIa is responsible for reduced inflammation observed in the colitis. The concentration of FVIIa used here in the treatment of colitis (250 µg/kg) was much lower than FVIIa concentrations required (4 to 10 mg/kg) to correct bleeding in hemophilia mice^28,63^. Furthermore, in earlier studies, we found no evidence that the administration of rFVIIa at 250 µg/kg enhances systemic coagulation in wild-type mice^38,64^. It is likely that the EPCR-FVIIa-induced signaling mechanism that suppresses inflammation and stabilizes barrier integrity could be responsible for the observed protection. Our observation that FVIIa fails to exert its protective effect in EPCR^−/−^ mice indicates EPCR-dependent FVIIa-mediated anti-inflammatory effects may be responsible for FVIIa’s protective effect in colitis. These data are consistent with our earlier reports that showed FVIIa elicits an anti-inflammatory effect via EPCR-dependent signaling^37,38,65^.

Intestinal epithelium undergoes cell renewal, and it is a constant process throughout adulthood in order to maintain mucosal homeostasis^66,67^. The intestinal stem cells, which exist at the base of intestinal crypts, maintains the self-renewing of the intestinal epithelium and also facilitates mucosal healing during colitis^66–68^. Hematopoietic stem cells (HSC) express EPCR^69,70^. The EPCR is often used as a marker for HSC, and EPCR was critical for homing and self-renewal of HSC in the bone marrow^69–71^. At present, it is unknown whether stem cells present at the base of intestinal crypts express EPCR. It is reasonable to hypothesize that the colon stem cells express EPCR, and EPCR expressing colon stem cells govern the mucosal homeostasis by regulating the self-renewal capacity of the colon stem cells. This could explain baseline abnormalities in the gut crypts of uninjured EPCR^−/−^ mice and severe colitis in DSS-treated EPCR^−/−^ mice.

In summary, our study provides direct evidence that EPCR expression in the colon modulates the pathogenesis of colitis by suppressing the activation of coagulation, leukocyte trafficking, and inflammation. Administration of pharmacological concentrations of rFVIIa reduces the severity of DSS-induced experimental colitis.

## Data Availability

The datasets generated during the current study are available from the corresponding author on reasonable request.
